# Frogs in the spotlight: a 16‐year survey of native frogs and invasive toads on a floodplain in tropical Australia

**DOI:** 10.1002/ece3.2237

**Published:** 2016-06-07

**Authors:** Gregory P. Brown, Richard Shine

**Affiliations:** ^1^ School of Life and Environmental Sciences, A08University of SydneySydneyNew South Wales2006Australia

**Keywords:** Amphibian populations, invasion ecology, time series, tropical ecology

## Abstract

Although widespread declines in anuran populations have attracted considerable concern, the stochastic demographics of these animals make it difficult to detect consistent trends against a background of spatial and temporal variation. To identify long‐term trends, we need datasets gathered over long time periods, especially from tropical areas where anuran biodiversity is highest. We conducted road surveys of four anurans in the Australian wet–dry tropics on 4637 nights over a 16‐year period. Our surveys spanned the arrival of invasive cane toads (*Rhinella marina*), allowing us to assess the invader's impact on native anuran populations. Our counts demonstrate abrupt and asynchronous shifts in abundance and species composition from one year to the next, not clearly linked to rainfall patterns. Typically, periods of decline in numbers of a species were limited to 1–2 years and were followed by 1‐ to 2‐year periods of increase. No taxa showed consistent declines over time, although trajectories for some species showed significant perturbations coincident with the arrival of toads. None of the four focal frog species was less common at the end of the study than at the beginning, and three of the species reached peak abundances after toad arrival. Survey counts of cane toads increased rapidly during the initial stage of invasion but have subsequently declined and fluctuated. Distinguishing consistent declines versus stochastic fluctuations in anuran populations requires extensive time‐series analysis, coupled with an understanding of the shifts expected under local climatic conditions. This is especially pertinent when assessing impacts of specific perturbations such as invasive species.

## Introduction

Dramatic declines of diverse taxa have led to concerns that we are in the midst of the Sixth Extinction Event (Eldredge 2001). As in previous Extinction Events, some phylogenetic lineages have experienced far higher rates of attrition than others. Anuran amphibians are often considered to be at disproportionately high risk (McCallum 2007; Wake and Vredenburg 2008). Many anuran species are declining rapidly due to habitat loss, climate change, pathogens, invasive species, among other threats and interactions (Kiesecker et al. 2001; Pounds et al. 2006).

Unfortunately, there are severe logistical impediments to documenting temporal changes in amphibian populations, because these animals often exhibit large spatial and temporal variation in abundance (Pechmann et al. 1991; Wake and Vredenburg 2008). That stochasticity makes it difficult to differentiate unusual declines from “background noise”. In turn, high demographic stochasticity in anuran populations is the outcome of at least two factors. First, these animals often have high fecundity, with clutch sizes in some families that are orders of magnitude greater than in amniote vertebrates (Wells 2010). Thus, a successful recruitment event can increase nonbreeding population size by a factor of thousands within a single breeding season. Second, most anurans depend on moist conditions for foraging and breeding, so that the timing and extent of rainfall can dramatically influence rates of survival, growth and reproduction (Todd et al. 2010). Untangling causal influences of threatening processes on anuran abundance thus requires long‐term datasets, gathered under standardized conditions (Dodd 2010); especially for the tropical habitats that contain a high proportion of anurans worldwide and are the sites of many catastrophic declines (McCallum 2007; Wake and Vredenburg 2008), few such datasets are available.

Amphibians are quintessential victims in the declining‐species narrative, but they can also play the role of villain. Invasive anurans such as cane toads (*Rhinella marina*, Linnaeus 1758), bullfrogs (*Lithobates catesbeianus*, Shaw 1802) and African clawed frogs (*Xenopus laevis*, Daudin 1802) have all been associated with declines in native species (Shine 2010; Snow and Witmer 2010; Lillo et al. 2011). The impact of cane toads on Australian ecosystems is the most intensively studied of these examples. In early research, cane toads were widely expected to have negative impacts on native anurans through direct predation, lethal poisoning and competition (Froggatt 1936; Van Dam et al. 2002; Shine 2014). Laboratory and field‐enclosure experiments have documented a diverse array of ecological interactions between cane toads and Australian frogs, within both aquatic and terrestrial phases of the life history (reviewed by Shine 2014). For example, thousands of tadpoles of native frogs are fatally poisoned when they attempt to ingest the toxic eggs of cane toads (Crossland et al. 2008) and metamorph frogs die when they try to eat metamorph toads (Greenlees et al. 2010); the presence of toads can modify activity levels and habitat selection of frogs (Greenlees et al. 2007; but see Bleach et al. 2014); and toads can transmit parasites to frogs (Pizzatto and Shine 2011) or take up parasite larvae that would otherwise infect frogs (Lettoof et al. 2013). Indirect ecological interactions may be even more important; for example, toads reduce predation pressure on frogs by virtually extirpating local populations of large carnivorous lizards (Brown et al. 2013b).

Cane toad invasion thus has the potential to affect frogs via several direct and indirect pathways, in both positive and negative ways. The net effect of toad arrival will reflect the combination of all of these pathways. Available data, mostly from short‐term surveys, suggest that the positive and negative effects cancel each other out, and hence that frog populations remain virtually unaffected by toad invasion (Shine 2014). That conclusion is highly counterintuitive, given the strength and diversity of mechanisms of impact, and might reflect a lack of power to detect toad impacts (because of stochastic demographics of anuran populations, as noted above). For an unequivocal result, we need long‐term datasets from standardized surveys, bracketing periods both before and after a potential perturbation (Crawford et al. 2010) such as the invasion of cane toads.

In the course of our long‐term ecological studies in the Australian wet–dry tropics, we have monitored abundance and species composition of the anuran fauna for 16 years. Seven years into that timeline, the cane toad invasion (which had been spreading westwards across tropical Australia since 1936) reached our study site. Our data thus provide an unusually robust opportunity not only to quantify the magnitude of natural temporal fluctuations in abundance of a tropical anuran assemblage, but also to explore the potential impact of an invasive anuran on its native counterparts. Our aims in the present study were threefold:

1. to document changes in cane toad abundance over the course of the invasion and establishment process,
2. to document long‐term temporal patterns of abundance of tropical anurans, and
3. to assess whether or not the arrival of cane toads has perturbed the abundance or activity patterns of native frogs.

## Materials and Methods

### Study area and species

**Figure 1:**
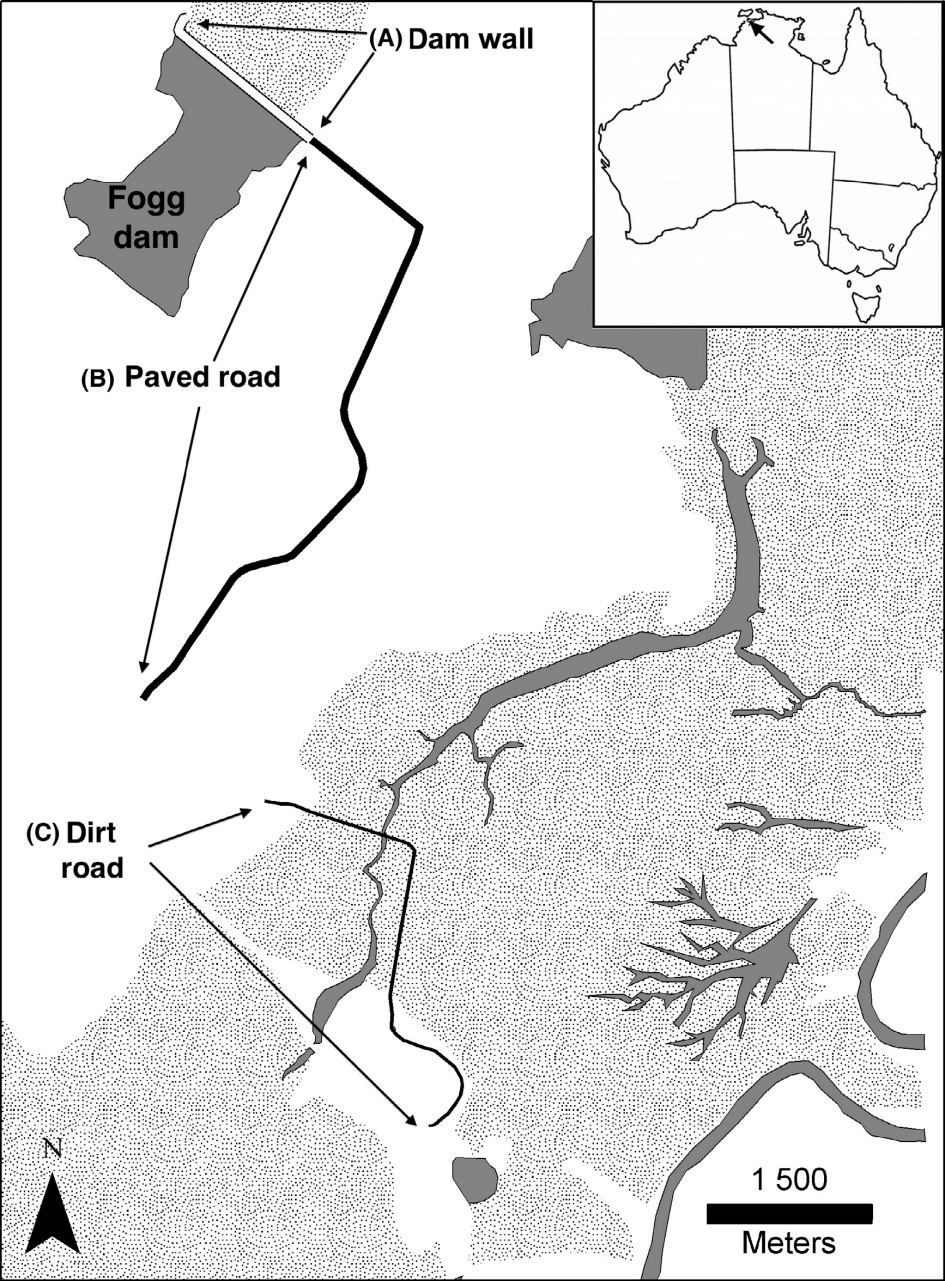
Map showing locations of anuran road‐survey transects. (A) Fogg Dam wall, (B) paved road, and (C) dirt road. Solid gray areas of the map indicate waterbodies, stippled gray areas represent floodplain, and white areas denote a mixture of agricultural land, pasture, and native woodland.

**Figure 2:**
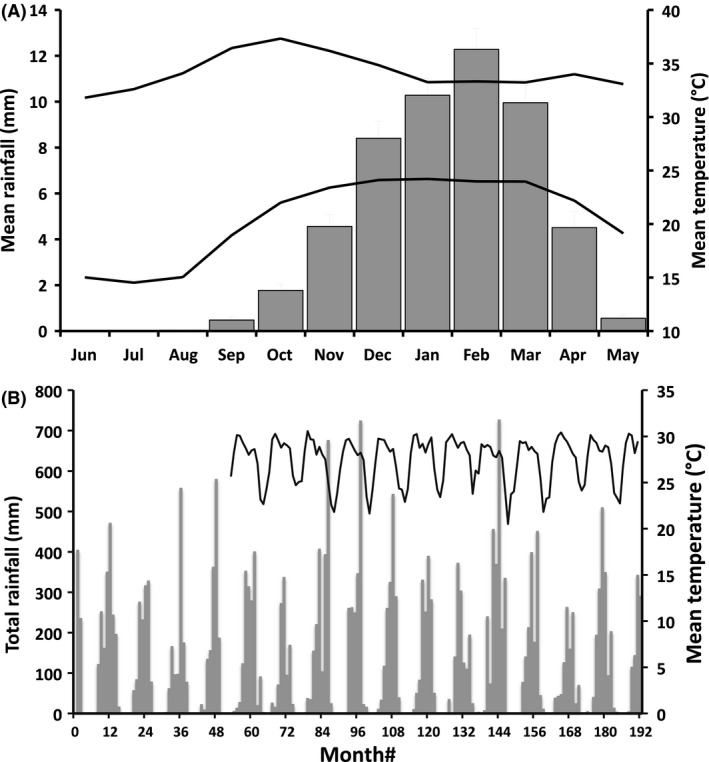
Graphical representation of the climate at our study site illustrating seasonal and long‐term variation. (A) Mean monthly values of rainfall (bars) and mean maximum and mean minimum temperatures. Note that *X*‐axis values begin in June each year in order to depict seasonal rainfall as a unimodal distribution. (B) Total monthly rainfall values (bars) and average monthly air temperatures over the 180‐month study period. Temperature data were not collected prior to August 2003.

The study took place in and around the Fogg Dam Conservation Reserve (12.58°S, 131.31°E; Fig. 1), 60 km southeast of the city of Darwin, in the Northern Territory of Australia. Frogs are abundant in the area, at least partly because *Batrachochytrium dendrobatidis*, the pathogen linked to declines in amphibian populations in other areas of Australia (Berger et al. 1998), has never been detected in the Northern Territory (www.bd-maps.net/maps/). Our study area is flat and low lying and is seasonally inundated by heavy monsoonal rain. The monsoons typically occur from December to February (Haynes et al. 1991), but with strong annual variation in the times of onset and cessation of flooding, and in the extent of inundation (Shine and Brown 2008). The temperature is high year round, with mean monthly temperatures usually >25°C (Fig. 2). Rainfall is sparse from May to October, with the total rainfall over this 6‐month period averaging <86 mm (of an annual mean around 1500 mm; see Fig. 2).

**Figure 3:**
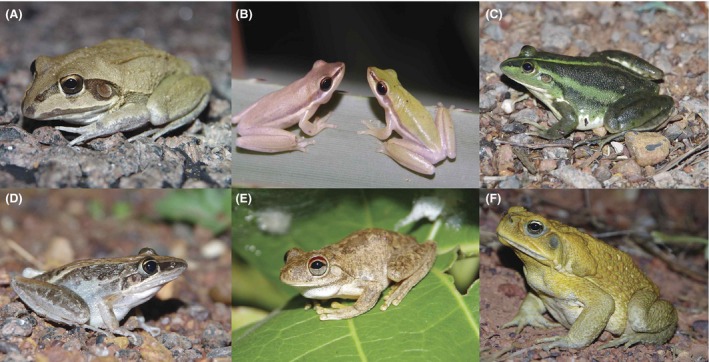
Photographs of the anuran species featured in our study, comprising five native frogs, (A) *Cyclorana australis*, (B) *Litoria bicolor*, (C) *L. dahlii*, (D) *L. nasuta*, (E) *L. rothii*, and the invasive cane toad, (F) *Rhinella marina*. Photo credit – G.P. Brown.

The study area contains approximately 20 anuran species (Davies and Tyler 1986), although many become inactive and sequester during the harshly desiccating conditions of the dry season (Tracy et al. 2007). We analyze encounter rates with five species of native frogs: *Cyclorana australis* (Gray 1842), *Litoria bicolor* (Gray 1842), *L*. *dahlii* (Boulenger 1896), *L. nasuta* (Gray 1842) and *L. rothii* (De Vis 1884) (Fig. 3). *Cyclorana australis* (giant burrowing frog) is a large (to 102 mm maximum body length: Davies and Tyler 1986) terrestrial frog that burrows and forms a cocoon during the dry season (Tracy et al. 2007). *Litoria bicolor* (northern dwarf tree frog) and *L. rothii* (Roth's tree frog) are small [to 29 mm and 57 mm, respectively (Davies and Tyler 1986)] arboreal frogs. *Litoria dahlii* [Dahl's aquatic frog, to 71 mm (Davies and Tyler 1986)] and *L. nasuta* [rocket frog, to 55 mm (Davies and Tyler 1986)], although in the “tree frog” genus *Litoria*, are semi‐aquatic and ground dwelling, respectively. Cane toads (to 230 mm) grow larger than any native Australian frogs and are exclusively terrestrial. However, toads overlap strongly in size, morphology and habits (including, dietary composition) with several native anuran species, notably *C. australis* (Greenlees et al. 2006, 2007).

### Survey methods

We conducted three sets of nocturnal surveys, for different purposes and of different durations.

#### Paved‐road survey of cane toads

This survey was established in 1998 to monitor snake numbers through a range of habitats (Brown et al. 2013a), but we incorporated counts of toads into the survey when they arrived in the study area. We counted all cane toads (but not frogs) from a slow‐moving car along the entire surface of a contiguous 9.8 km length of paved roadway, including the road atop the Fogg Dam wall (Fig. 1). Toads were first seen in the study area in February 2005, and this survey was conducted on 2878 subsequent nights until the end of February 2015 (79% of nights over that period). Although toads were known to be in the study area in February 2005, they did not begin to appear in road surveys until November 2005.

#### Dirt‐road survey of cane toads and *Cyclorana australis*

This survey was conducted as part of a study comparing microhabitat use between cane toads and the native frog species most similar to toads in morphology and ecology, *C. australis*. For the present paper, we use data from this survey to assess whether counts of *C. australis* have changed over the period of toad residency. The survey was initiated in 2007, 2 years after toad arrival, and was carried out opportunistically on a total of 94 nights over five wet seasons: 13 nights in 2007, 36 nights in 2008, 23 nights in 2009 and 11 nights in 2010. The survey was not conducted between 2011 and 2013 but was resumed for 11 nights during 2014. The survey was carried out from a slow‐moving (10 km/h) all‐terrain cycle along a 4.4 km dirt road running through forest and floodplain habitat on a pastoral property (Beatrice Hill Farm; Fig. 1). We counted each toad and *C. australis* along this transect on each survey night.

#### Grid‐based survey of native frogs and cane toads at Fogg Dam

Our most comprehensive survey of native anurans began as an adjunct to a study on the ecology of frog‐eating snakes. We conducted regular counts of native frogs in 10 survey grids, marked with paint on the surface of the road that runs atop the wall of Fogg Dam (Fig. 1). The 10 grids were equally spaced across the 1500 m length of the dam wall and each one enclosed a 2 × 3 m area of the road surface. Counts were made approximately 1 h after dark, on foot or from a slow‐moving vehicle, on a total of 4637 nights between March 1999 and February 2015 (79% of all nights over that 192‐month period).

### Analysis

As an initial straightforward assessment of anuran population trends, we performed Spearman nonparametric correlations of survey counts for each species against year #. Because we began our surveys in the month of March, we based the year # used in nonparametric correlations on 12‐month periods beginning in March and ending in February.

We used time‐series intervention analyses to assess temporal trends in native frog counts before and after the arrival of cane toads. This multiple regression approach uses an autocorrelated error structure to accommodate the cyclical nature of data collected over seasons and years and can include explanatory covariates such as weather or search effort. It also incorporates a segmented regression structure to model temporal patterns in counts before and after an “intervention event” (e.g., arrival of cane toads) as well as the step change in counts immediately coincident with the “event” (Huitema and Mckean 2000; Piegorsch and Bailer 2005). These processes are modeled as regression parameters as follows:

$$\begin{array}{cc} Y= & B0(\text{intercept})+B1(\text{time})+B2(\text{intervention})\\ & +B3(\text{time since intervention})+B4(\text{covariate})\\ & +B5(\text{covariate})+\text{autocorrelated error} \end{array}$$

In the present instance, the dependent variable (*Y*) is the mean monthly count of each frog species. We ln‐transformed count data (after adding 0.05 to each value) to meet normality assumptions. “Time” is a continuous variable denoting the month of the study (1–192), and “intervention” is a dummy variable used to differentiate pretoad (months 1–80 = “0”) from posttoad (months 81–192 = “1”) periods. “Time since intervention” is a continuous variable with a value of 0 until the first month after the intervention and thenceforth taking on values of the number of months elapsed since the intervention. We used total monthly rainfall and the number of nights surveyed in each month as a covariate in our analyses of frog survey counts. Thus, *B0* is the intercept, *B1* is the pretoad slope, *B2* is the step change in frog counts upon the arrival of toads, *B3* is the change in slope from the pretoad to posttoad period, *B4* is the parameter for the effect of rainfall on mean monthly frog counts, and *B5* is the parameter for the effect of survey effort. More details on the implementation of this analysis to assess cane toad impacts on animal survey data can be found in Brown et al. (2011b) and Price‐Rees et al. (2010).

## Results

### Paved‐road survey of cane toads

**Figure 4:**
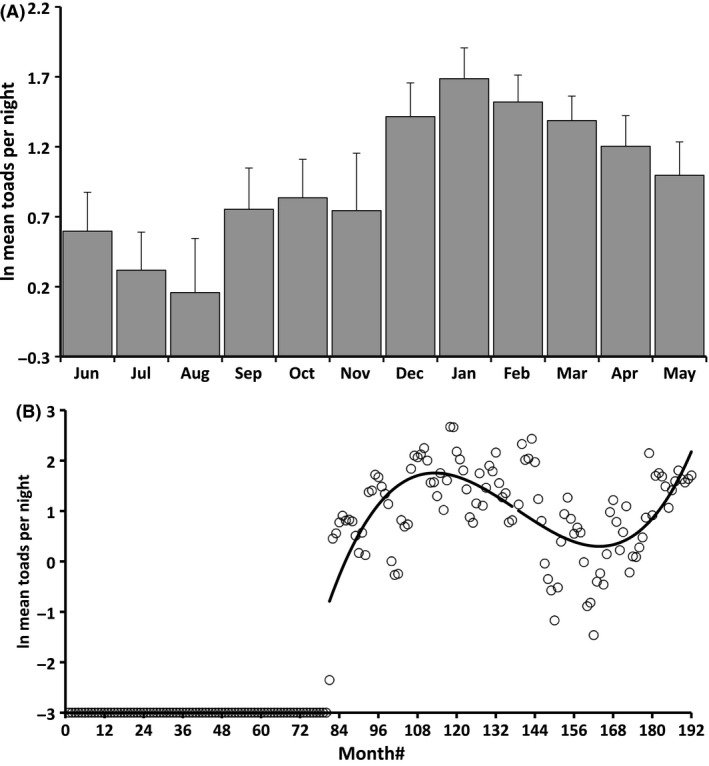
Temporal variation in encounter rates of cane toads on 9.8 km of road during nocturnal surveys conducted on 2878 nights following the toads' first arrival in 2005. (A) Monthly mean values illustrating the extent of seasonality. (B) Mean values by month over the course of the study, illustrating changes in time since arrival. Line is a cubic function fitted by regression.

Toad counts differed among months (*F*_11,99_ = 3.09, *P *=* *0.0013; Fig. 4A), with more encounters during the wet season. Toad numbers increased dramatically over the first 3–4 years postinvasion, declined over the next 3 years and then began to climb again in 2013 (Fig. 4B). A cubic polynomial regression with autocorrelated error structure produced significant linear, quadratic and cubic time effects (all |*t*| > 7.28, df = 104, all *P *<* *0.0001). A simple nonparametric correlation of mean annual counts versus year # reveals a nonsignificant time trend over the 10 years that toads have been present at the study site (Spearman *r *=* *0.10, *P *=* *0.77).

### Dirt‐road survey of cane toads and *Cyclorana australis*

**Figure 5:**
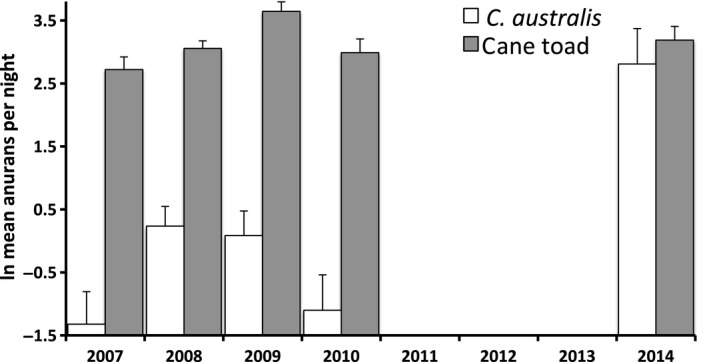
Annual variation in dirt road‐survey counts of *Cyclorana australis* (white bars) and cane toads (gray bars) during five wet seasons over an 8‐year period. Numbers of *C. australis* increased substantially in the most recent survey.

The numbers of *C. australis* were 20 times higher during the final year of the survey than during the first year (Fig. 5). A two‐way ANOVA on anuran counts, with species (toad vs. *C. australis*) and year # as factors, produced a significant interaction term (*F*_4,178_ = 6.93, *P *<* *0.0001). During the first 4 years of the survey, *C. australis* were much less commonly recorded than were toads, but during the final year of the survey, the numbers of *C. australis* and toads were similar (Fig. 5).

### Grid‐based survey of native frogs and cane toads at Fogg Dam

We counted a total of 5786 anurans over 4637 nights, with up to 84 anurans observed simultaneously occupying a single 2 × 3 m survey grid. Four focal *Litoria* species dominated the survey observations, together composing 94% of sightings (*Litoria bicolor* 30%, *L. dahlii* 40%, *L. nasuta* 20% and *L. rothii* 4%). After their arrival, cane toads represented 1.6% of all anurans counted in survey grids. Other less common anuran species encountered in the survey grids were *L. rubella* (total *N *=* *19), *L. dorsalis* (*N *=* *103), *L. caerulea* (*N *=* *3), *L. tornieri* (*N *=* *2), *Limnodynastes convexiusculus* (*N *=* *23), *Platyplectrum ornatum* (*N *=* *3), *Cyclorana australis* (*N *=* *9) and *C. longipes* (*N *=* *5).

**Figure 6:**
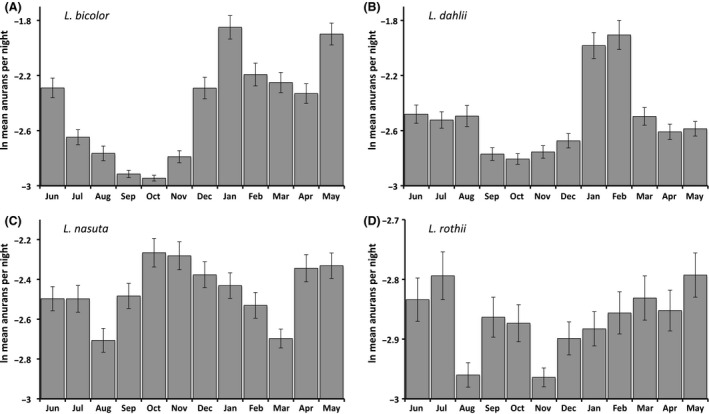
Monthly mean values of grid survey counts of native frogs at Fogg Dam, illustrating seasonal variation. Surveys were conducted on 4637 nights between March 1999 and February 2015. Data are shown for (A) *L. bicolor*, (B) *L. dahlii*, (C) *L. nasuta*, and (D) *L. rothii*. Differences in *Y*‐axis scaling among species reflect differences in their relative abundance in the surveys.

Despite the highly seasonal rainfall, *L. rothii* was equally common in our surveys among all months of the year (*F*_11,179_ = 1.41, *P *=* *0.17; Fig. 6A). Numbers of *L. nasuta,* in contrast, varied significantly among months (*F*_11,179_ = 1.96, *P *=* *0.035), but not in a manner consistent with rainfall patterns. Wet and dry seasons both included months with high and low *L. nasuta* numbers (Fig. 6A). In contrast, *L. dahlii* and *L. bicolor* were more common during wet‐season months (both *F*_11,179_ > 2.16, both *P *<* *0.019; Fig. 6A). Simple correlations suggested that annual rainfall had little impact on the number of amphibians observed in a given year. Based on the mean annual values (12‐month periods from March to February), we found no significant correlations between rainfall and counts of any of the four frog species (*N *=* *16, all Spearman |*r*| < 0.25, all *P *>* *0.33). Although the number of nights surveyed varied from 233 to 337 among the 16 years, only counts of *L. nasuta* were related to survey effort (Spearman *r *=* *0.55, *P *=* *0.027). The average counts of the other three frog species were independent of the number of surveys (all |*r*| < 0.49, all *P *>* *0.06; Table 1). Effects of rainfall and survey effort on frog counts became more evident when these factors were used as covariates in the intervention analysis regressions (see below).

**Table 1:** Mean (± standard error) annual (March to February) encounter rates (anurans per night) of four native frog species (all in the genus *Litoria*) surveyed in grids atop the Fogg Dam wall, and for cane toads (*Rhinella marina*) surveyed along a 9.8 km section of road. Shaded rows indicate surveys conducted after the arrival of cane toads in 2005

Year #	Year	No. of nights	*L. dahlii*	*L. bicolor*	*L. nasuta*	*L. rothii*	*R. marina*
1	1999–2000	277	0.37 ± 0.132	0.15 ± 0.066	0.24 ± 0.05	0.02 ± 0.006	–
2	2000–2001	313	0.22 ± 0.047	0.27 ± 0.08	0.26 ± 0.08	0.03 ± 0.006	–
3	2001–2002	315	0.09 ± 0.027	0.21 ± 0.06	0.22 ± 0.052	0.01 ± 0.004	–
4	2002–2003	316	0.01 ± 0.007	0.76 ± 0.224	0.29 ± 0.08	0.01 ± 0.004	–
5	2003–2004	302	0.12 ± 0.049	0.87 ± 0.243	0.65 ± 0.149	0.1 ± 0.026	–
6	2004–2005	337	0.56 ± 0.305	0.44 ± 0.132	0.32 ± 0.067	0.06 ± 0.019	–
7	2005–2006	287	0.61 ± 0.251	0.27 ± 0.098	1.06 ± 0.334	0.04 ± 0.013	0.45 ± 0.235
8	2006–2007	327	0.76 ± 0.575	0.37 ± 0.103	0.19 ± 0.032	0.07 ± 0.018	2.78 ± 0.44
9	2007–2008	276	2.56 ± 1.762	0.24 ± 0.084	0.11 ± 0.018	0.17 ± 0.061	3.50 ± 0.761
10	2008–2009	279	0.38 ± 0.126	0.09 ± 0.026	0.12 ± 0.038	0.05 ± 0.013	7.41 ± 1.11
11	2009–2010	268	2.47 ± 2.093	0.11 ± 0.039	0.04 ± 0.016	0.03 ± 0.014	4.94 ± 0.607
12	2010–2011	251	0.17 ± 0.077	0.12 ± 0.024	0.08 ± 0.019	0.04 ± 0.019	5.74 ± 0.963
13	2011–2012	292	0.03 ± 0.013	0.3 ± 0.11	0.13 ± 0.032	0.01 ± 0.004	1.66 ± 0.325
14	2012–2013	293	0.000	0.43 ± 0.156	0.1 ± 0.037	0.000	1.21 ± 0.285
15	2013–2014	265	0.03 ± 0.025	0.35 ± 0.112	0.2 ± 0.039	0.07 ± 0.02	2.25 ± 0.600
16	2014–2015	239	0.17 ± 0.138	0.67 ± 0.259	0.14 ± 0.051	0.09 ± 0.023	4.92 ± 0.246

Nonparametric correlations between frog abundance and year number indicated a significant decline in counts of *L. nasuta* over the 16 years (Spearman *r *=* *−0.60, *P *=* *0.013; Table 1). Correlations between time and numbers of the other three frog species were nonsignificant (*L. bicolor*, Spearman *r *=* *−0.02, *P *=* *0.94; *L. dahlii, r *=* *−0.30, *P *=* *0.26; *L. rothii, r *=* *0.20, *P *=* *0.45).

**Figure 7:**
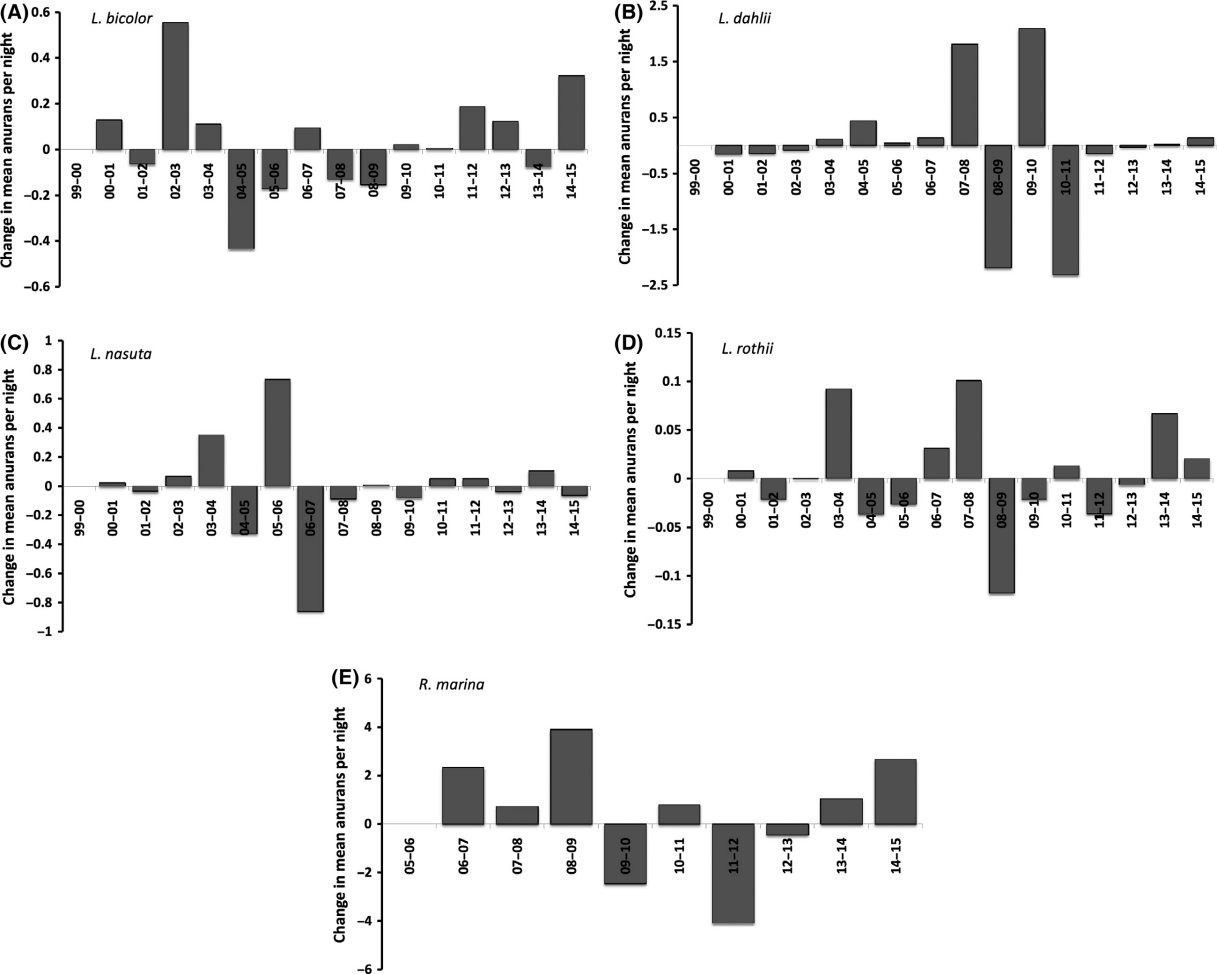
Between‐year changes in mean counts for four native frog species encountered in grid survey counts at Fogg Dam during 4637 nights between March 1999 and February 2015. (A) *L. bicolor*, (B) *L. dahlii*, (C) *L. nasuta*, and (D) *L. rothii*. Cane toads (E) were enumerated during surveys along 9.8 km of road between November 2005 and February 2015. For all species, episodes of continued increase or decrease longer than 2 years were rare.

In these four frog species, changes in mean encounter rate from 1 year to the next tended to be modest. In all species, however, occasional explosive and asynchronous increases occurred. These dramatic increases typically were followed by equally dramatic decreases (to a more “usual” level) the following year (Fig. 7). Overall, the common dynamic was modest fluctuation from year to year, with successive increases then decreases (consecutive increases or decreases lasting more than 2 years were uncommon, Fig. 7). The lack of synchrony in annual count fluctuations among the four native frogs is evidenced by the lack of correlation between pairs of species in the extent of change from the previous year (all Spearman |*r*| < 0.50, all *P *>* *0.06). Similarly, there was no significant consistency in the direction of changes in counts (increase vs. decrease) over successive years between pairs of species (all *χ* ^2^ < 1.73, all *P *>* *0.18). Nonetheless, significant correlations did exist between pairs of frog species in mean counts (as opposed to change in counts). Average annual counts of *L. bicolor* and *L. nasuta* were positively correlated with each other (*N *=* *16, Spearman *r *=* *0.52, *P *=* *0.040), and counts of *L. dahlii* and *L. bicolor* were negatively correlated with each other (*N *=* *16, Spearman *r *=* *−0.59, *P *=* *0.017). Thus, although counts of some frog species tended to be high at the same time, they did not increase (or decrease) in unison. Commonly, one species was declining at the same time as other species were increasing.

### Changes in counts of native frogs after the invasion of cane toads

**Figure 8:**
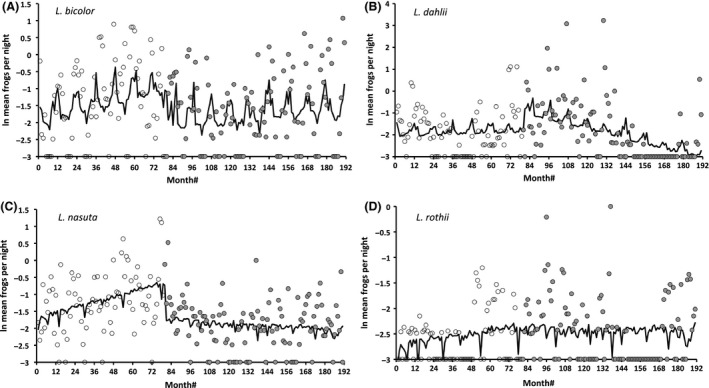
Temporal changes in mean monthly grid survey counts of native frogs at Fogg Dam, over 192 months (beginning in March 1999) encompassing the arrival of cane toads. Open symbols indicate mean values prior to cane toad invasion (which occurred in November 2005, month #81), and shaded symbols indicate counts subsequent to toad invasion. Lines are predicted relationships from intervention analysis using monthly rainfall and # of survey nights as covariates. Data are shown for (A) *L. bicolor*, (B) *L. dahlii*, (C) *L. nasuta*, and (D) *L. rothii*. Differences in *Y*‐axis scaling among species reflect differences in their relative abundance in the surveys.

High variance among the 192 mean monthly counts over the 16‐year period tends to obscure general patterns in anuran numbers (Fig. 8; note that the *Y*‐axes are log‐transformed), but major temporal changes are evident nonetheless. For example, between mid‐2011 and mid‐2013 (months #150–170), two of the focal species (*L. dahlii*,* L. rothii*) virtually disappeared from survey grids for 20 consecutive months, before reappearing in late 2013 (Fig. 8). Numbers of the other common species (*L. bicolor, L. nasuta*) remained at normal levels over the same period (Fig. 8).

Numbers of *L. dahlii* fluctuated more than did those of the other frog species and this variation was partly attributable to levels of rainfall each month. *Litoria dahlii* numbers were at maximum levels 2–3 years after toads arrived, but after a further 2–3 years they temporarily disappeared from surveys (see above; Table 2, Fig. 8). This decline over the posttoad period is evident from the significant slope‐change parameter in the intervention analysis.

**Table 2:** Results of intervention analysis on the effects of cane toad invasion on survey counts of four native frog species

Species	Test statistic	Time	Step	Slope change	Rainfall	Survey nights
*L. dahlii*	*t*	0.30	1.71	−**2.16**	**2.26**	−0.58
	*P*	0.76	0.09	**0.032**	**0.025**	0.57
*L. bicolor*	*t*	1.90	−**2.99**	−0.86	**4.27**	1.62
	*P*	0.06	**0.0032**	0.39	**<0.0001**	0.11
*L. nasuta*	*t*	**2.36**	−**3.29**	−**2.33**	−0.07	1.86
	*P*	**0.019**	**0.0012**	**0.021**	0.94	0.06
*L. rothii*	*t*	0.91	−0.15	−0.67	0.18	**3.43**
	*P*	0.37	0.88	0.50	0.86	**0.0007**

Columns represent independent variables from a multiple regression, and values represent the test statistics and significance levels for each factor. “Time” is a linear trend over the 192‐month study. “Step” models a change in survey count level coinciding with the time of toad arrival (Nov 2005, month #81). “Slope change” represents the degree to which the overall time trend changed trajectory subsequent to toad arrival. “Rainfall” represents variation in survey counts attributable to monthly levels of rainfall. “Survey nights” is a covariate to quantify search effort. Boldface font indicates significant values.

Counts of *L. bicolor* were strongly linked to monthly rainfall patterns and exhibited a detectable downward step change coincident with toad arrival (Table 2, Fig. 8). Counts of *L. rothii* were higher during months when more surveys were conducted, but were unaffected by monthly rainfall and unaffected by the arrival of toads (Table 1, Fig. 8).

The strongest evidence for a negative impact of cane toad invasion comes from *L. nasuta* (Table 1, Fig. 8). Over the first 80 months of surveys, *L. nasuta* numbers were increasing and reached their maximum level immediately prior to the arrival of toads. Upon the arrival of toads, numbers of *L. nasuta* dropped and subsequently fluctuated around an intermediate level. Counts of *L. nasuta* were not significantly affected by monthly rainfall, but were marginally affected by the number of survey nights (*P *=* *0.06).

## Discussion

Our method of surveying amphibians (counts on roads) has weaknesses as well as strengths. First, our use of direct counts as a measure of abundance is less robust than are estimates based on mark–recapture or distance sampling (Schmidt and Pellet 2009). Counts are influenced by anuran behavior as well as underlying abundance; for example, the arrival of cane toads may have influenced frog counts not only by changing frog numbers, but also because frogs tend to avoid toads (Greenlees et al. 2007; Narayan et al. 2013). Given that toads constituted <2% of anurans surveyed on the wall of Fogg Dam, however, any such effect will have been minor. Second, we only sampled a proportion of the native frog species in our study area, focusing on floodplain rather than woodland taxa. Such biases are inevitable; to have included the full diversity of anuran taxa would have required a far greater diversity of survey methods, and it was logistically impractical. The strength of our surveys lies in their duration, their temporal regularity, and the high, consistent probability of detecting an anuran sitting on the surface of a road.

### Temporal changes in toad numbers

Counts of toads along the paved‐road transect showed dramatic seasonal cycles. During the driest months (June, July, August), toads were usually only seen atop the wall of Fogg Dam (adjacent to permanent water) rather than along sections of the road passing through farmland and woodland. This seasonality is attributable to the toads' dependence on a source of moisture during the dry season. During the wet season, when the landscape is uniformly moist, toads can move about freely; but when water is scarce, their activity is limited to areas with a dependable source of moisture (Brown et al. 2011a).

Toad numbers in our study area peaked 3–4 years after the initial invaders arrived, declined over the next 3–4 years, and then began to increase again. This “traveling wave” pattern is commonly observed in species invasions (Simberloff and Gibbons 2004; Hilker et al. 2005). Density‐dependent factors (such as food shortage and disease) likely cause these declines (Freeland 1986; Freeland et al. 1986; Simberloff and Gibbons 2004). Now, almost 10 years after toads arrived at our study site, their numbers may begin to fluctuate stochastically in a manner similar to that of native frogs. Future transient increases in toad numbers presumably will not reach levels as high as the initial peak observed early in the invasion.

### Temporal changes in native frog numbers

Our counts of native frogs are biased in several ways, for example toward floodplain dwellers and species that do not avoid open areas. Notably, one of the most abundant floodplain frog species at our study site rarely appeared in our survey grids. Based on calls and observations made away from the road, the marbled frog, *Limnodynastes convexiusculus*, is extremely common at Fogg Dam. However, this species restricts its activity to vegetated areas at the road margin and avoids the open surface of the road. When they cross the road, they do so with alacrity (G. P. Brown, pers. obs.). Species that linger on the road surface are more likely to be seen and counted in the survey grids (Beckmann and Shine 2015).

Peaks in frog counts on the wall of Fogg Dam coincide with seasonal migrations linked to reproduction. Early in the wet season (December–January) as water levels rise, frogs cross the road from the dam to the newly inundated floodplain. The floodplain may offer a better habitat for tadpole development, with fewer predators (Cabrera‐Guzmán et al. 2015) and thus be preferred for reproduction. At the end of the wet season (April–May), the migration occurs in reverse: both adult frogs and newly metamorphosed juveniles leave the floodplain and cross the road back to the permanent water. These seasonal movements create bimodal peaks each year and are detectable in the fitted lines in Figure 8.

Frog counts commonly changed two‐ or threefold from year to year, but such changes were rarely consistent for more than two consecutive years. Explosive increases from 1 year to the next likely are attributable to high breeding success (Alford and Richards 1999), such that metamorph and juvenile frogs appeared in the survey grids as they migrated from the floodplain to the permanent water of the dam. Between successive years, encounter rates with native frogs tended to decline approximately as often as they increased. This may be a commonplace pattern among amphibians, although the proportion of annual declines versus increases may vary among families according to life‐history traits such as fecundity and offspring survival (Alford and Richards 1999).

The lack of interspecific synchrony in year‐to‐year changes in counts of native frogs suggests that activity and/or population size are not determined by any common external factor. A simple scenario such as “years with abundant rain are good for frogs” is clearly inapplicable, because counts of the different species do not peak in unison. Frog numbers may indeed depend upon rainfall, but (if so) the schedule of rainfall that maximizes survival/recruitment/activity must differ among the species that we studied.

### Impact of cane toads on native frogs

To understand global patterns of amphibian declines, we need to examine populations that are not declining as well as those which are (Pechmann et al. 1991). Our intervention analyses indicated that after the arrival of cane toads, survey counts changed in three frog species. The nature of the perturbations varied among species, from a drop in mean encounter rates immediately after toad arrival (*L. bicolor*) through to a decrease in the slope of the relationship between count and time between pre‐ and posttoad periods (*L. dahlii*), or both of these changes (*L. nasuta*). Despite these alterations in survey count trajectories subsequent to toad arrival, none of our focal frog species was less common at the end of the study than at the beginning. In addition, peak counts for three frog species (*L. bicolor*,* L. dahlii*, and *L. rothii*) occurred subsequent to the arrival of toads. These results exemplify the difficulty of using temporal shifts to attribute a causal relationship between toad arrival and changes in frog abundance.

Although intervention analysis is a useful tool for quantifying changes in temporal trends in response to a perturbation at a known time, it cannot distinguish correlation from causation. Because our study was correlational rather than experimental, we cannot rule out the possibility that changes in frog counts were instead (or additionally) affected by some unknown factor that occurred at about the same time that toads reached our study site (Wagner et al. 2002; Gilmour et al. 2006). This caveat is important. Although we have used intervention analysis to quantify authentic toad‐related impacts (Price‐Rees et al. 2010), we have also detected spurious “impacts” as a result of events occurring coincident with toad invasion. For example, intervention analysis indicated a strong negative impact of toad invasion on one snake species (water python, *Liasis fuscus*: Brown et al. 2011b). In a pattern similar to *L. nasuta* in the present study, numbers of water pythons were increasing prior to toad arrival but declined thereafter. The decline in python numbers was not due to toads, however; instead, it was due to an unusual rainfall event (soon after the arrival of toads) that inundated the Adelaide River floodplain and thus drowned the python's rodent prey (Ujvari et al. 2011a,b).

The example of water pythons shows that any relationship between toad arrival and the abundance of native species could be an artifact of factors such as the timing of rainfall events (including the flooding event described above). Plausibly, changes in frog numbers are affected by such factors as well. Regardless, the clear conclusion from our extensive dataset is that the impacts of cane toad invasion on the four focal frog species have been minor overall: current counts of abundance of these species are well within the range of pretoad fluctuations. Importantly however, our four focal frog species represent a minority of anuran taxa in the study area, and we cannot address the extent to which toads may have impacted other species. Information on toad impacts on less abundant, more specialized and taxonomically diverse frog species is critical to properly assess the threat posed by the invasive species.

Although our survey data on *C. australis* along the dirt‐road transect are shorter term and lack a pretoad baseline, they show that this species too has increased in the presence of toads. Plausibly, the observed increase in *C. australis* counts could be an indirect effect of the negative impact toads have on the predators of frogs. In a previous study, 13 of 22 *C. australis* radiotracked at a nearby site were eaten by varanid (monitor) lizards (Tracy et al. 2007). The toxin of cane toads is lethal to these giant lizards (Brown et al. 2013b), and toads caused a 95% decline in the abundance of *Varanus panoptes* at the site where we surveyed *C. australis* (Brown et al. 2013b). The increased abundance of *C. australis* as toads arrived thus may reflect the removal of a major reptilian predator by cane toads.

Despite the long time frame and intensive sampling effort of the current study, we are left with few clear‐cut conclusions about the causal factors underpinning temporal shifts in anuran abundance in this system. We have shown that the numbers of native frogs change considerably from one year to the next and tend to do so asynchronously. If local weather drives such changes, it must affect different species in subtly different ways. The overall pattern is dynamic and changeable, without strong overt links to abiotic factors. Likewise, the arrival of a large invasive anuran had relatively little overall impact on the dynamics of this floodplain anuran assemblage. In a system such as this, any conclusions about overall trends in species abundance or conservation status must be tentative unless supported by long‐term datasets on underlying stochasticity, and (ideally) an understanding of the effects of abiotic factors on faunal numbers and composition.

## Conflict of Interest

None declared.

## References

[ece32237-bib-0001] Alford, R. A. , and S. J. Richards . 1999 Global amphibian declines: a problem in applied ecology. Annu. Rev. Ecol. Syst. 30:133–165.

[ece32237-bib-0002] Beckmann, C. , and R. Shine . 2015 Do the numbers and locations of road‐killed anuran carcasses accurately reflect impacts of vehicular traffic? J. Wildl. Manag. 79:92–101.

[ece32237-bib-0003] Berger, L. , R. Speare , P. Daszak , D. E. Green , A. A. Cunningham , C. L. Goggin , et al. 1998 Chytridiomycosis causes amphibian mortality associated with population declines in the rain forests of Australia and Central America. Proc. Natl. Acad. Sci. 95:9031–9036. 9671799 10.1073/pnas.95.15.9031 PMC21197

[ece32237-bib-0004] Bleach, I. , C. Beckmann , G. P. Brown , and R. Shine . 2014 Effects of an invasive species on refuge‐site selection by native fauna: the impact of cane toads on native frogs in the Australian tropics. Austral Ecol. 39:50–59.

[ece32237-bib-0005] Brown, G. P. , C. Kelehear , and R. Shine . 2011a Effects of seasonal aridity on the ecology and behaviour of invasive cane toads in the Australian wet‐dry tropics. Funct. Ecol. 25:1339–1347.

[ece32237-bib-0006] Brown, G. P. , B. L. Phillips , and R. Shine . 2011b The ecological impact of invasive cane toads on tropical snakes: field data do not support laboratory‐based predictions. Ecology 92:422–431. 21618921 10.1890/10-0536.1

[ece32237-bib-0007] Brown, G. P. , M. J. Greenlees , B. L. Phillips , and R. Shine . 2013a Road transect surveys do not reveal any consistent effects of a toxic invasive species on tropical reptiles. Biol. Invasions 15:1–11.

[ece32237-bib-0008] Brown, G. P. , B. Ujvari , T. Madsen , and R. Shine . 2013b Invader impact clarifies the roles of top‐down and bottom‐up effects on tropical snake populations. Funct. Ecol. 27:351–361.

[ece32237-bib-0009] Cabrera‐Guzmán, E. , M. R. Crossland , D. Pearson , J. K. Webb , and R. Shine . 2015 Predation on invasive cane toads (*Rhinella marina*) by native Australian rodents. J. Pest. Sci. 88:143–153.

[ece32237-bib-0010] Crawford, A. J. , K. R. Lips , and E. Bermingham . 2010 Epidemic disease decimates amphibian abundance, species diversity, and evolutionary history in the highlands of central Panama. Proc. Natl. Acad. Sci. 107:13777–13782. 20643927 10.1073/pnas.0914115107 PMC2922291

[ece32237-bib-0011] Crossland, M. R. , G. P. Brown , M. Anstis , C. M. Shilton , and R. Shine . 2008 Mass mortality of native anuran tadpoles in tropical Australia due to the invasive cane toad (*Bufo marinus*). Biol. Conserv. 141:2387–2394.

[ece32237-bib-0012] Davies, M. , and M. J. Tyler . 1986 Frogs of the northern territory. Conservation Commission of the Northern Territory, Darwin.

[ece32237-bib-0013] Dodd, C. K. 2010 Amphibian ecology and conservation: a handbook of techniques. Oxford University Press, Oxford.

[ece32237-bib-0014] Eldredge, N. 2001 The sixth extinction. *An ActionBioscience. org original article. American Institute of Biological Sciences* [Online.] http://endangeredink.com/programs/population_and_sustainability/extinction/pdfs/Eldridge-6th-extinction.pdf.

[ece32237-bib-0015] Freeland, W. J. 1986 Populations of cane toad *Bufo marinus* in relation to time since colonization. Aust. Wildl. Res. 13:321–330.

[ece32237-bib-0016] Freeland, W. J. , B. L. J. Delvinqueir , and B. Bonnin . 1986 Food and parasitism of the cane toad, Bufo‐Marinus, in relation to time since colonization. Aust. Wildl. Res. 13:489–499.

[ece32237-bib-0017] Froggatt, W. W. 1936 The introduction of the great Mexican Toad *Bufo marinus* into Australia. Aust. Nat. 9:163–164.

[ece32237-bib-0018] Gilmour, S. , L. Degenhardt , W. Hall , and C. Day . 2006 Using intervention time series analyses to assess the effects of imperfectly identifiable natural events: a general method and example. BMC Med. Res. Methodol. 6:16. 16579864 10.1186/1471-2288-6-16 PMC1481564

[ece32237-bib-0019] Greenlees, M. , G. Brown , J. Webb , B. Phillips , and R. Shine . 2006 Effects of an invasive anuran [the cane toad (Bufo marinus)] on the invertebrate fauna of a tropical Australian floodplain. Anim. Conserv. 9:431–438.

[ece32237-bib-0020] Greenlees, M. J. , G. P. Brown , J. K. Webb , B. L. Phillips , and R. Shine . 2007 Do invasive cane toads (*Chaunus marinus*) compete with Australian frogs (*Cyclorana australis*)? Austral Ecol. 32:900–907.

[ece32237-bib-0021] Greenlees, M. J. , B. L. Phillips , and R. Shine . 2010 Adjusting to a toxic invader: native Australian frogs learn not to prey on cane toads. Behav. Ecol. 21:966–971.

[ece32237-bib-0022] Haynes, C. , M. Ridpath , and M. A. Williams . 1991 Monsoonal Australia: landscape, ecology and man in northern lowlands. Balkema A. A., Rotterdam.

[ece32237-bib-0023] Hilker, F. , M. Lewis , H. Seno , M. Langlais , and H. Malchow . 2005 Pathogens can slow down or reverse invasion fronts of their hosts. Biol. Invasions 7:817–832.

[ece32237-bib-0024] Huitema, B. E. , and J. W. Mckean . 2000 Design specification issues in time‐series intervention models. Educ. Psychol. Measur. 60:38–58.

[ece32237-bib-0025] Kiesecker, J. M. , A. R. Blaustein , and L. K. Belden . 2001 Complex causes of amphibian population declines. Nature 410:681–684. 11287952 10.1038/35070552

[ece32237-bib-0026] Lettoof, D. C. , M. J. Greenlees , M. Stockwell , and R. Shine . 2013 Do invasive cane toads affect the parasite burdens of native Australian frogs? Int. J. Parasitol. Parasites Wildl. 2:155–164. 24533330 10.1016/j.ijppaw.2013.04.002 PMC3862496

[ece32237-bib-0027] Lillo, F. , F. P. Faraone , and M. L. Valvo . 2011 Can the introduction of *Xenopus laevis* affect native amphibian populations? Reduction of reproductive occurrence in presence of the invasive species. Biol. Invasions 13:1533–1541.

[ece32237-bib-0028] McCallum, M. L. 2007 Amphibian decline or extinction? Current declines dwarf background extinction rate. J. Herpetol. 41:483–491.

[ece32237-bib-0029] Narayan, E. J. , J. F. Cockrem , and J.‐M. Hero . 2013 Sight of a predator induces a corticosterone stress response and generates fear in an amphibian. PLoS One 8:e73564. 24009756 10.1371/journal.pone.0073564 PMC3757005

[ece32237-bib-0030] Pechmann, J. H. K. , D. E. Scott , R. D. Semlitsch , J. P. Caldwell , L. J. Vitt , and J. W. Gibbons . 1991 Declining amphibian populations: the problem of separating human impacts from natural fluctuations. Science 253:892–895. 17751826 10.1126/science.253.5022.892

[ece32237-bib-0031] Piegorsch, W. W. , and A. J. Bailer . 2005 Analyzing environmental data. John Wiley & Sons, Chichester.

[ece32237-bib-0032] Pizzatto, L. , and R. Shine . 2011 Ecological impacts of invading species: do parasites of the cane toad imperil Australian frogs? Austral Ecol. 36:954–963.

[ece32237-bib-0033] Pounds, A. J. , M. R. Bustamante , L. A. Coloma , J. A. Consuegra , M. P. L. Fogden , P. N. Foster , et al. 2006 Widespread amphibian extinctions from epidemic disease driven by global warming. Nature 439:161–167. 16407945 10.1038/nature04246

[ece32237-bib-0034] Price‐Rees, S. J. , G. P. Brown , and R. Shine . 2010 Predation on toxic cane toads (*Bufo marinus*). may imperil bluetongue lizards (*Tiliqua scincoides intermedia*, Scincidae) in tropical Australia. Wildl. Res. 37:166–173.

[ece32237-bib-0035] Schmidt, B. R. , and J. Pellet . 2009 Quantifying abundance: counts, detection probabilities, and estimates Pp. 465–479 *in* Amphibian ecology and conservation: a handbook of techniques. Dodd C. K., Jr , ed. Oxford University Press, Oxford.

[ece32237-bib-0036] Shine, R. 2010 The ecological impact of invasive cane toads (*Bufo marinus*) in Australia. Q. Rev. Biol. 85:253–291. 20919631 10.1086/655116

[ece32237-bib-0037] Shine, R. 2014 A review of ecological interactions between native frogs and invasive cane toads in Australia. Austral Ecol. 39:1–16.

[ece32237-bib-0038] Shine, R. , and G. P. Brown . 2008 Adapting to the unpredictable: reproductive biology of vertebrates in the Australian wet‐dry tropics. Philos. Trans. R. Soc. Lond. B Biol. Sci. 363:363–373. 17638689 10.1098/rstb.2007.2144 PMC2606755

[ece32237-bib-0039] Simberloff, D. , and L. Gibbons . 2004 Now you see them, now you don't! Population crashes of established introduced species. Biol. Invasions 6:161–172.

[ece32237-bib-0040] Snow, N. , and G. Witmer . 2010 American bullfrogs as invasive species: a review of the introduction, subsequent problems, management options, and future directions. Pp. 86–89 *in* Proceedings of the 24th Vertebrate Pest Conference. Timm R. M. and Fagerstone K. A. eds.

[ece32237-bib-0041] Todd, B. D. , D. E. Scott , J. H. Pechmann , and J. W. Gibbons . 2010 Climate change correlates with rapid delays and advancements in reproductive timing in an amphibian community. Proc. R. Soc. B Biol. Sci., 278:2191–2197. doi: 10.1098/rspb.2010.1768 10.1098/rspb.2010.1768 PMC3107619 21159681

[ece32237-bib-0042] Tracy, C. R. , S. J. Reynolds , L. McArthur , C. R. Tracy , and K. A. Christian . 2007 Ecology of aestivation in a cocoon‐forming frog, *Cyclorana australis* (Hylidae). Copeia 2007:901–912.

[ece32237-bib-0043] Ujvari, B. , R. Shine , L. Luiselli , and T. Madsen . 2011a Climate‐induced reaction norms for life‐history traits in pythons. Ecology 92:1858–1864. 21939082 10.1890/11-0129.1

[ece32237-bib-0044] Ujvari, B. , R. Shine , and T. Madsen . 2011b How well do predators adjust to climate‐mediated shifts in prey distribution? A study on Australian water pythons. Ecology 92:777–783. 21608485 10.1890/10-1471.1

[ece32237-bib-0045] Van Dam, R. A. , D. Walden , and G. W. Begg . 2002 A preliminary risk assessment of cane toads in Kakadu National Park. Supervising Scientist, Darwin, NT, Australia, Scientist Report 164.

[ece32237-bib-0046] Wagner, A. K. , S. B. Soumerai , F. Zhang , and D. Ross‐Degnan . 2002 Segmented regression analysis of interrupted time series studies in medication use research. J. Clin. Pharm. Ther. 27:299–309. 12174032 10.1046/j.1365-2710.2002.00430.x

[ece32237-bib-0047] Wake, D. B. and V. T. Vredenburg . 2008 Are we in the midst of the sixth mass extinction? A view from the world of amphibians. Proc. Natl. Acad. Sci. 105:11466–11473. 18695221 10.1073/pnas.0801921105 PMC2556420

[ece32237-bib-0048] Wells, K. D. . 2010 The ecology and behavior of amphibians. University of Chicago Press, Chicago.

